# Supplementary material for: The Adhesion GPCR ADGRL2 engages Gα13 to Enable Epidermal Differentiation
Source: bioRxiv. 2025 Apr 14:2025.02.19.639154. Preprint. [Version 3] doi: 10.1101/2025.02.19.639154 (PMC11888183; doi:10.1101/2025.02.19.639154)

bioRxiv preprint doi: https://doi.org/10.1101/2025.02.19.639154; this version posted April 14, 2025. The copyright holder for this preprint (which was not certified by peer review) is the author/funder. This article is a US Government work. It is not subject to copyright under 17 USC 105 and is also made available for use under a CC0 license.

Fig. S1. CRISPR-Flow and Perturb-seq GPCR knockout screens of epidermal GPCRs in differentiating keratinocytes. **a**, Numbers of gene targets and sgRNAs in the CRISPR plasmid library. **b**, Selected keratinocyte progenitor and differentiation genes. **c**, sgRNA count distribution in plasmid library. **d**, CRISPR-Flow gating strategy. **e**, Quantitative flow cytometric analysis of KRT10 expression changes from progenitors to differentiated cells. **f**, Perturb-seq mRNA overall knockout efficiency of target sgRNAs populations compared to non-target sgRNA populations. **g**, Heatmap visualization of Perturb-seq results; columns correspond to mRNAs while rows indicate the effect of individual gene perturbation on progenitor and differentiation genes.

Fig. S2. ADGRL2 affects keratinocyte differentiation in 2D and organoid models. **a**, Temporal heatmap illustrating GPCR mRNA expression during across days 0 to 6 of calcium-induced differentiation of primary human keratinocytes in vitro. **b**, ADGRL2 knockdown evaluation by Western blot. **c**, Heatmap of significant altered genes, FDR<0.05 with fold change >2 or <0.5, upon ADGRL2 knockdown. **d**, Quantification of KRT10, TGM1, Ki67 immunofluorescent signal in ADGRL2 knockdown organoid models, unpaired t Test. **e**, Western blot of the mosaic samples. **f**, Quantification of KRT10 signal in mosaic tissue control and L2 siRNAs. In **f**, paired t Test, n = 11.

Fig. S3. ADGRL2 activates Gα13 subtype to enable epidermal differentiation. **a**, Cellular localization of HA tagged ADGRL2 full-length, ADGRL2-CTF, PAR1-ADGRL2, EK-ADGRL2, ADGRL2-CTF-Δ7TA proteins in HEK293T cells evaluated by immunostaining. **b**, Western blot of ADGRL2-CTF and ADGRL2-CTF-Δ7TA protein expression. **c**, SRE-RF luciferase reporter measurements of ADGRL2-CTF and ADGRL2 TA peptide-deficient mutants. **d**, Gα proteins Perturb-seq showing enrichment of knock-out cells along the pseudotime trajectory (Mann-Whitney log10 P) compared to cells with safe target guides. A positive enrichment in low pseudotime cells indicates nominates a gene as necessary for differentiation. **e**, GNA13 knockdown evaluation by Western blot. **f**, RNA-Seq analysis of GNA13 knockdown samples sourced from two independent skin donors, showing all genes with log2 fold change >0.2 or <-0.2, red/blue genes are the ones upregulated/downregulated during differentiation, white genes are unchanged ones. **g**, Heatmap of significant altered genes, FDR<0.05 with fold

bioRxiv preprint doi: https://doi.org/10.1101/2025.02.19.639154; this version posted April 14, 2025. The copyright holder for this preprint (which was not certified by peer review) is the author/funder. This article is a US Government work. It is not subject to copyright under 17 USC 105 and is also made available for use under a CC0 license.

change >2 or <0.5, upon GNA13 knockdown. **h**, Gene Ontology analysis of all significantly altered mRNAs (FDR<0.05) by GNA13 knockdown, based on RNA-seq data. **i**, Correlation between GNA13 and ADGRL2 knockdown RNA-seq results. **j**, Quantification of KRT10, TGM1 immunofluorescent signal in *GNA13* depleted regenerated human skin organoid tissue, unpaired t Test.

Fig. S4. Cryo-EM analysis of the ADGRL2-Gα13 complex in lipid nanodiscs. a, The receptor and Gα13 constructs used in the study. Sequence corresponding to the CTF of ADGRL2 (TA and 7TM) was inserted after a hemagglutinin signal peptide (HA) and a methionine residue. The plasmid for expressing the miniGα13heterotrimer is the same as for the ADGRL3- Gα13 complex[37]. **b**, Size-exclusion chromatography (SEC) profile and SDS-PAGE of purified miniGα13-coupled ADGRL2. **c**, Representative 2D class averages of the ADGRL2- Gα13 complex. **d**, Cryo-EM data processing workflow for the ADGRL2-Gα13 complex in lipid nanodiscs. **e**, Angular distribution heat map of particles used for the global 3D reconstruction of the ADGRL3-Gα13 complex. **f**, Gold-standard Fourier shell correlation (FSC) curves of the locally refined receptor and miniGα13 reconstructions. **g**, Cryo-EM map of the ADGRL2-Gα13 complex in lipid nanodiscs showing the resolved density for two cholesterol molecules (colored in blue). The first methionine residue (M) used in the receptor construct was also resolved. **h**, Cryo-EM density and models are shown for TMs 1-7, the tethered agonist (TA) peptide and the CLR molecules of the ADGRL2-Gα13 complex. **i**, Binding mode of the TA peptide, and the bent TM6 and TM7 (highlighted by dashed lines) in the miniGα13-coupled ADGRL2. **j**, Interactions between ICL2 and the miniGα13 α5 helix of ADGRL2. Key residues are shown as sticks. **k**, Comparison of the basal Gα13 activity of ADGRL2-CTF WT and ICL2 mutants (F943A and V942/F943A) by titrating different amounts of transfected plasmids by BRET2 assay. **l**, Cellular localization of HA tagged ADGRL2-CTF-F943A and V942/F943A mutants in HEK293T cells evaluated by immunostaining. **m**, Western blot of empty vector, ADGRL2-CTF, ADGRL2-CTF-F943A and ADGRL2-CTF-V942/F943A in HEK293T cells. **n**, Unsharpened Cryo-EM map of the ADGRL2-Gα13 complex showing that ICL3 of the receptor protrudes from the lipid bilayer to interact with miniGα13. **o**, The model and EM density for the interface between ICL3 of the receptor and the QQQ patch of Gα13. **p**,

bioRxiv preprint doi: https://doi.org/10.1101/2025.02.19.639154; this version posted April 14, 2025. The copyright holder for this preprint (which was not certified by peer review) is the author/funder. This article is a US Government work. It is not subject to copyright under 17 USC 105 and is also made available for use under a CC0 license.

Structural comparison of ADGRL2 and ADGRL3 when coupled with miniGα13, highlighting extracellular regions. **q**, Western blot of Gα13 wild-type and AAA mutant in HEK293T cells.

bioRxiv preprint doi: https://doi.org/10.1101/2025.02.19.639154; this version posted April 14, 2025. The copyright holder for this preprint (which was not certified by peer review) is the author/funder. This article is a US Government work. It is not subject to copyright under 17 USC 105 and is also made available for use under a CC0 license.

Figure S1

a

Categories	Gene numbers	gRNAs
GPCR	101	404
Keratinocytes control genes	22	88
Progenitor control genes	22	88
Non-target gRNAs	NA	40
Total	145	620

b

Keratinocyte differentiation associated genes

IRF6	GRHL3	MAF	MCM5	PKM	DUT
ZNF750	POU2F3	MAFB	ITGB1	PFN1	FEN1
KLF4	JUNB	VDR	FERMT1	DNMT1	ARPC1B
CEBPA	PRDM1	TP63	CD151	YWHAZ	DDIT4
EHF	OVOL1	YAP1	EGFR	S100A8	
NOTCH1	TFAP2A	PRMT1	DNAJC9	MGST1	
NOTCH2	HOPX	CALML3	UBE2S	ITGAV	
RBPJ	MPZL3	NOTCH3	SFN	TYMS	

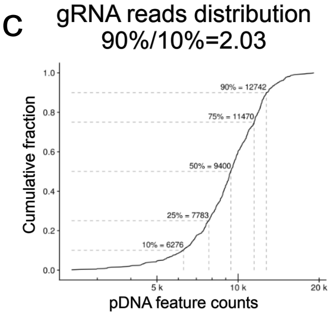

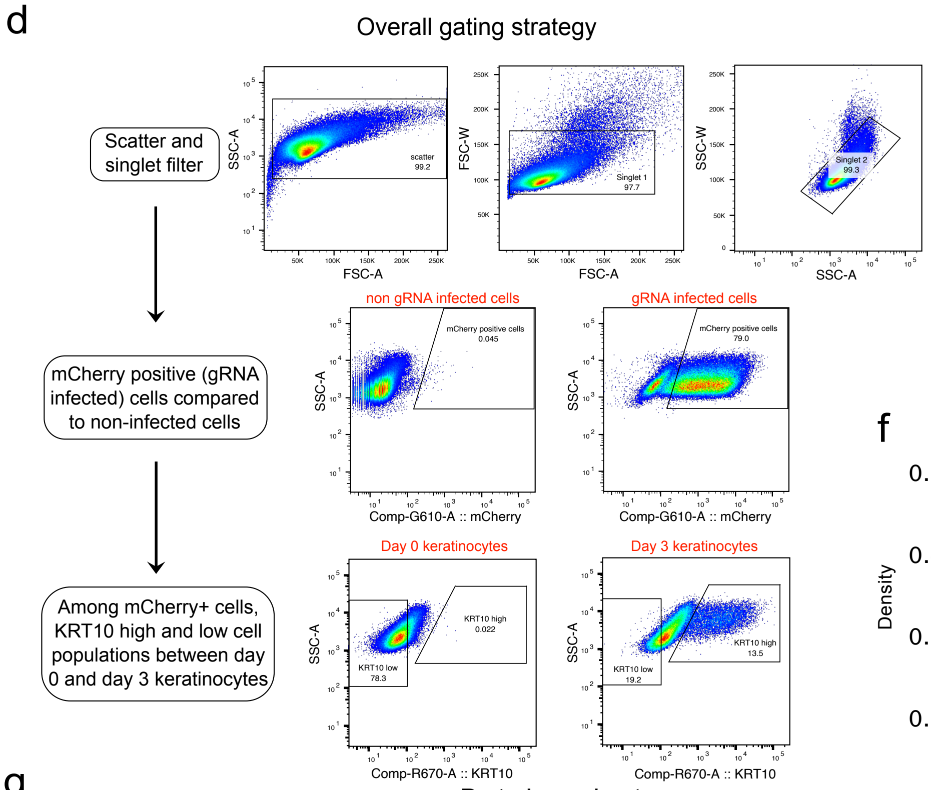

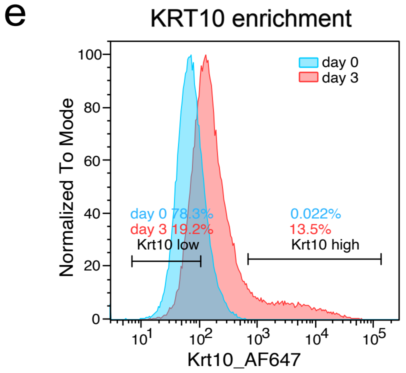

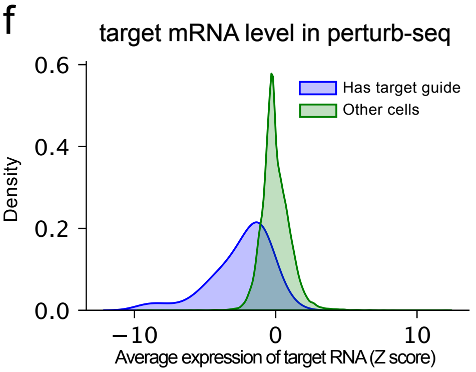

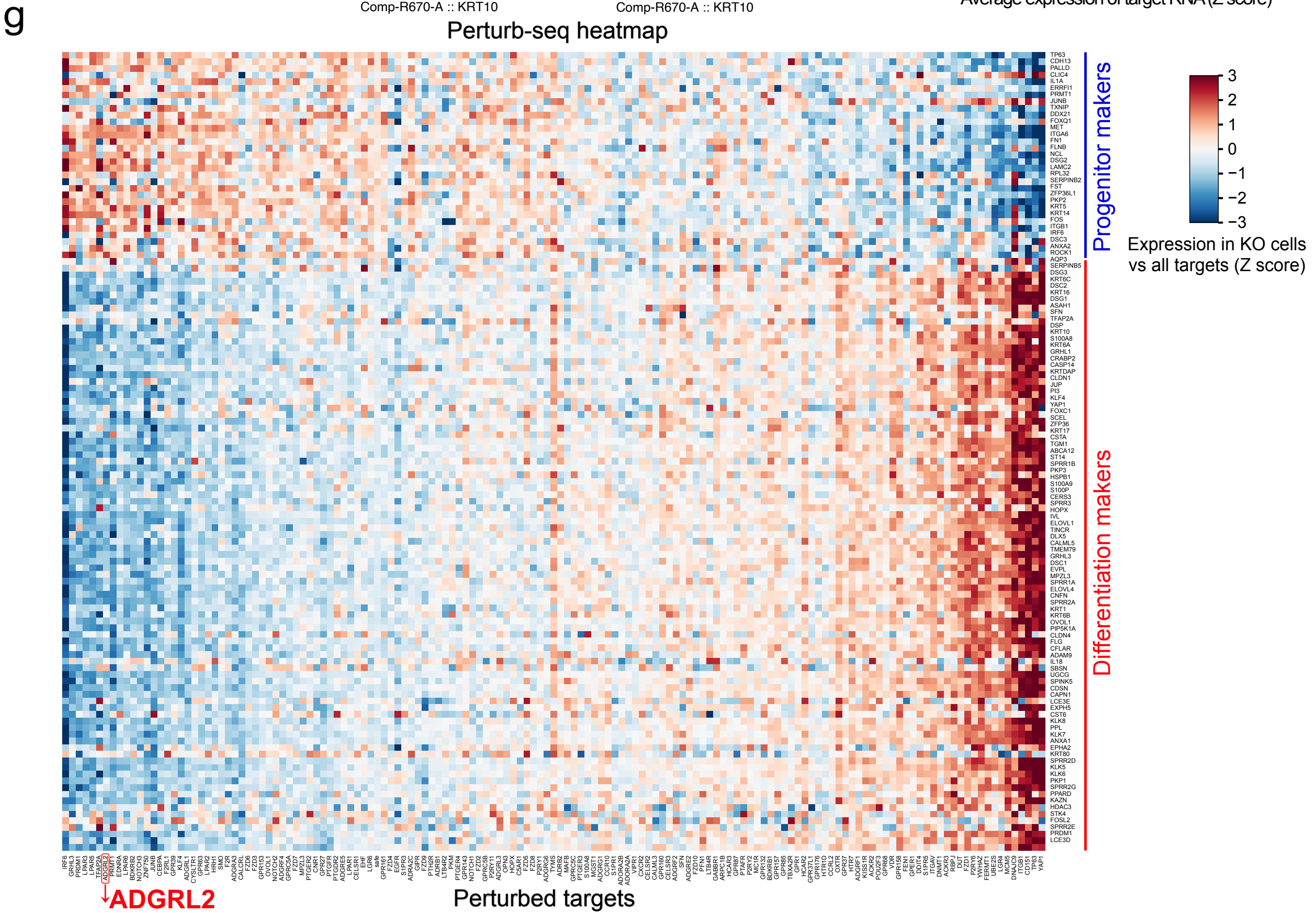

bioRxiv preprint doi: https://doi.org/10.1101/2025.02.19.639154; this version posted April 14, 2025. The copyright holder for this preprint (which was not certified by peer review) is the author/funder. This article is a US Government work. It is not subject to copyright under 17 USC 105 and is also made available for use under a CC0 license.

Figure S2

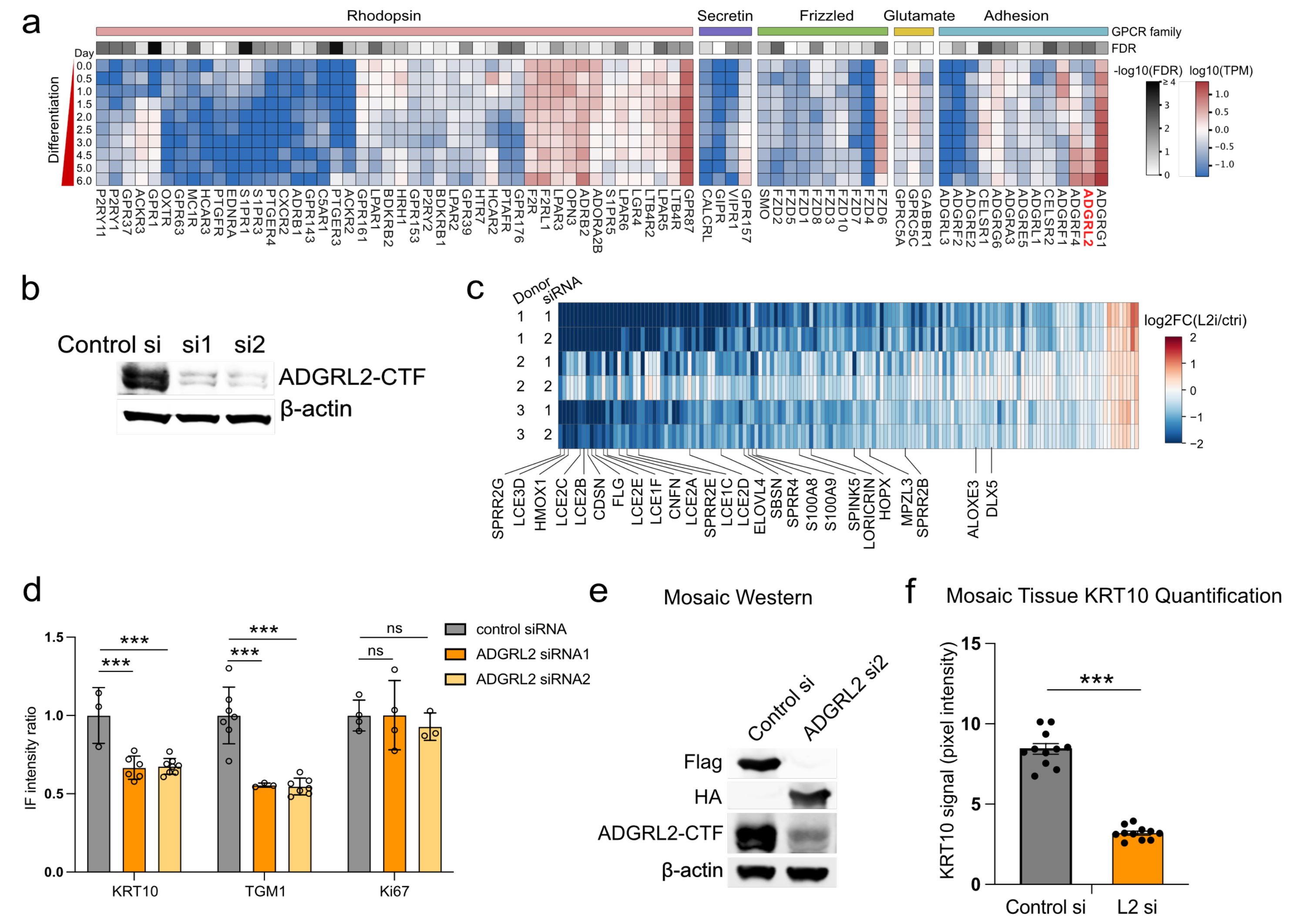

bioRxiv preprint doi: https://doi.org/10.1101/2025.02.19.639154; this version posted April 14, 2025. The copyright holder for this preprint (which was not certified by peer review) is the author/funder. This article is a US Government work. It is not subject to copyright under 17 USC 105 and is also made available for use under a CC0 license.

Figure S3

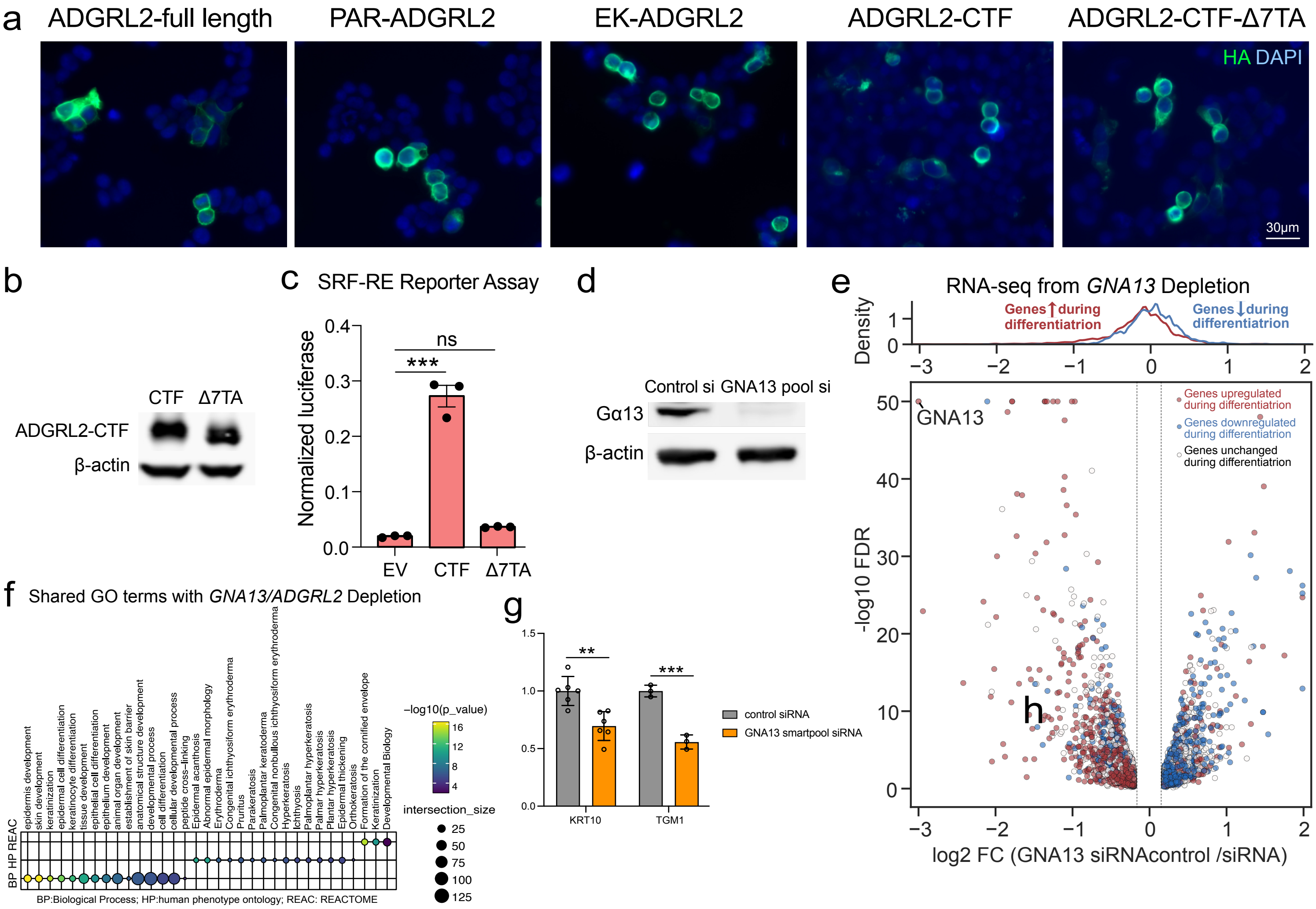

bioRxiv preprint doi: https://doi.org/10.1101/2025.02.19.639154; this version posted April 14, 2025. The copyright holder for this preprint (which was not certified by peer review) is the author/funder. This article is a US Government work. It is not subject to copyright under 17 USC 105 and is also made available for use under a CC0 license.

Figure S4

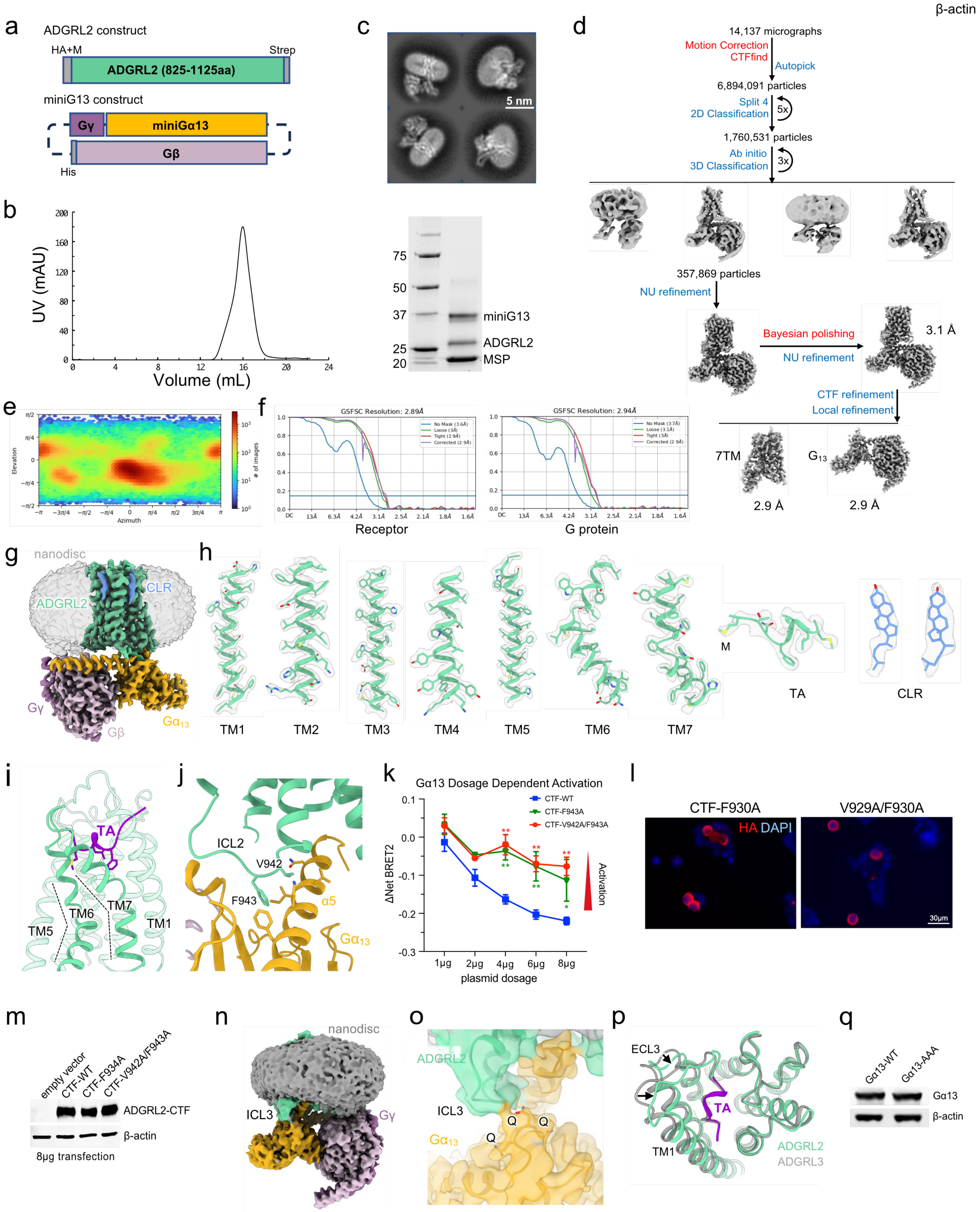

Supplement: 1 [file NIHPP2025.02.19.639154V3-supplement-1.pdf]
